# Within‐leaf variation in embolism resistance is not a rule for compound‐leaved angiosperms

**DOI:** 10.1002/ajb2.16447

**Published:** 2024-12-16

**Authors:** Ian M. Rimer, Scott A. M. McAdam

**Affiliations:** ^1^ Department of Botany and Plant Pathology Purdue University West Lafayette IN USA

**Keywords:** embolism, palmately compound, pinnately compound, pulvinus, rachis, segmentation, xylem

## Abstract

**Premise:**

Hydraulic segmentation, caused by the difference in embolism resistance across plant organs, provides a sacrificial layer of cheaper plant organs, like leaves, to protect more costly organs, such as stems, during drought. Within‐leaf hydraulic segmentation has been observed in two compound‐leaved tree species, with leaflets being more vulnerable than the rachis or petiole. Many herbaceous species have compound leaves, and some species have leaflets that are associated with pulvini at the base of the lamina, which could provide an anatomical means of preventing embolism from spreading within a leaf because of the higher number of vessel endings in the pulvinus.

**Methods:**

We used the optical vulnerability method to investigate whether differences in embolism resistance were observed across the leaf tissues of six herbaceous species and one deciduous tree species with compound leaves. Our species selection included both palmately and pinnately‐compound leaved species, one of each with a pulvinus at the base of the leaflets.

**Results:**

We found considerable variation in embolism resistance across the species measured, but no evidence of variation in embolism resistance within the leaf. In two species with pulvini, we observed major embolism events crossing the pulvinus, spreading from the rachis or petiole into the lamina, and embolizing both tissues at the same water potential.

**Conclusions:**

We conclude that within‐leaf hydraulic segmentation, caused by variation in embolism resistance, is not a universal phenomenon to compound‐leaved species and that the presence of a pulvinus does not provide a barrier to embolism spread in compound leaves.

Water transport in plants is driven by evaporation at the leaf surface, which causes an extreme pressure gradient from roots to leaves in the xylem conduits (Tyree and Sperry, 1989). When water availability is limited, such as during drought, the pressure gradient in the xylem can reach critical thresholds, allowing air to enter the xylem conduits, forming an embolism (Cardoso et al., 2020; Kaack et al., 2021; Avila et al., 2022), leading to plant death (Skelton et al., 2018; Cardoso et al., 2020; Herbette et al., 2021). Given the lethality of extensive embolism formation in most plants, some species protect critical organs by developing a series of more vulnerable downstream tissues in the water transport system (Zimmerman, 1983; Tyree and Ewers, 1991), commonly described as hydraulic segmentation (Sperry and Love, 2015; Choat et al., 2018). Hydraulic segmentation is primarily thought of as a safety mechanism to preserve the integrity of the stem xylem through the sacrificial loss of highly vulnerable leaves or smaller, more vulnerable, stems during drought (Wolfe et al., 2016). There have been many reports of the leaves of a species being less resistant than the stem (Tyree and Alexander, 1993; Tyree et al., 1993; Joyce and Steiner, 1995; Nardini and Pitt, 1999; Pivovaroff et al., 2014; Charrier et al., 2016; Johnson et al., 2016; Levionnois et al., 2020). There are also reports of no hydraulic segmentation between leaves and stems, particularly in herbaceous species (Skelton et al., 2017), unless secondary growth forms in the stem (Skelton et al., 2017; Jin et al., 2019; Li et al., 2020; Guan et al., 2022; Haverroth et al., 2024). There are even some reports of reverse segmentation, whereby leaves are more embolism‐resistant than stems, and under some environmental conditions, it is predicted that this reverse segmentation may be adaptively relevant (Alnus and Sauter, 1996; Way et al., 2013; Wilkening et al., 2023).

Variation in embolism resistance is commonly observed across the venation network of leaves (Brodribb et al., 2016; Scoffoni et al., 2017). In leaves, embolism often initiates in the xylem conduits of the midrib or petiole, propagating into the minor veins at lower water potentials (Scoffoni and Jansen, 2016; Scoffoni et al., 2017; Hochberg et al., 2019; Albuquerque et al., 2020; Brodribb et al., 2021; Petruzzellis et al., 2023; Jain et al., 2024). If embolism is artificially induced in the petiole, it will rapidly spread throughout the xylem of the leaf (Guan et al., 2021), which suggests that embolism formation in the leaf is a function of the connectivity of the leaf venation network as well as water potential. Embolism in the midrib severs the water supply to minor veins and is associated with partial or complete leaf death during drought (Cardoso et al., 2020; Brodribb et al., 2021; Tonet et al., 2023; Jain et al., 2024).

While imaging studies find that the midrib and, by extension, the petiole contain the most vulnerable xylem of entire leaves, a recent report has found an opposite, yet considerable, hydraulic segmentation between the rachis and the lamina of the compound leaves of deciduous trees (Song et al., 2022). In the compound leaves of *Juglans* and *Fraxinus*, the midribs of leaflets are the first to embolize during dehydration, yet these embolism events do not extend into the rachis (Song et al., 2022). Song et al. (2022) have found that leaflets are more vulnerable to embolism than the rachis of the compound leaves, which in turn are more vulnerable than the stem. The inhibition of embolism spread from leaflets to rachis in compound leaves is hypothesized to be due to the occurrence of an abscission zone at the base of the leaflets in these species, which provides an additional safety mechanism for preventing embolism spread.

Many species have elaborate compound leaves, with abscission zones (Osborne and Morgan, 1989) or a pulvinus (Rodrigues and Machado, 2008) at the base of the leaflets. Both the pulvinus and leaflet abscission zones could serve as barriers to embolism propagation across the leaf because of the complex xylem anatomy in these regions. Pulvini are joint‐like structures that serve as an active motor organ (Moran, 2007; Song et al., 2014; Wang et al., 2023) for nastic movements of leaflets (Fromm and Lautner, 2007; Uehlein and Kaldenhoff, 2008). The vascular system in the pulvinus often divides into multiple, radially symmetric bundles through the region (Nakata and Takahara, 2022), and the xylem conduits are often very short compared to conduits outside of the pulvinus (Fleurat‐Lessard, 1988). The short conduits through pulvini could result in a high concentration of pit membranes, increasing interconduit connectivity (Rodrigues and Machado, 2008), but inhibiting the spread of embolism through this zone. This inhibition could reduce the speed of embolism propagation (Kaack et al., 2021), facilitating apparent hydraulic segmentation between the rachis or petiole, and leaf lamina. In many species, the attachment points of leafets to the rachis or petiole are also the site of an abscission zone that activates during drought, facilitating leaflet shedding and reduced transpiration (Van Doom and Stead, 1997; Wang et al., 2023). Hydraulic segmentation in compound leaves would add an additional layer of protection to preserve the structural integrity of more costly stem tissue. It is not yet known whether the variation in embolism resistance observed across the compound leaves of deciduous trees (Song et al., 2022) is indicative of a major selective pressure for the evolution of compound leaves and is thus a phenotype observed in all species with compound leaves.

In this study, we used the optical vulnerability method (Brodribb et al., 2017), to determine if within‐leaf hydraulic segmentation is a rule in pinnately and palmately compound‐leaved species. We hypothesize that like in the compound leaves of deciduous trees (Song et al., 2022), hydraulic segmentation occurs across the compound leaves of all angiosperm species and is especially pronounced in species with pulvini at the base of leaflets, which could act as a barrier to embolism spread from the lamina to the rachis, resulting in a more embolism‐resistant rachis or petiole compared to leaf lamina. To test this, we simultaneously examined the spread of embolism through petiole, rachis, and leaflets in four pinnately‐ and three palmately‐compound leaved species, one of each with a pulvinus and one with an abscission zone at the base of the leaflets (Table 1). Species were chosen to represent a diversity of pinnately‐ and palmately‐compound leaved species with variable drought resistance, frost tolerance, and phenology. These species included *Chamaecrista fasciculata*, which is an autumn‐flowering, North American native, herbaceous, prairie species; *Eranthis hyemalis*, which is introduced to North America and is a late‐winter flowering geophyte; *Cardamine hirsuta* and *Oxalis debilis*, which are invasive, spring‐flowering, herbs in North America; *Ailanthus altissima*, which is a globally‐invasive, deciduous tree; and *Petroselinum crispum* and *Trifolium repens*, which are biennial and perennial, cultivated, herbaceous species, respectively.

**Table 1:** Description of the herbaceous species used to investigate hydraulic segmentation across compound leaves, including family, type of compound leaf, and presence or absence of pulvini.

Species	Family	Compound	Pulvini	Abscission Zone
*Cardamine hirsuta* L.	Brassicaceae	*Pinnately*	‐	‐
*Chamaecrista fasciculata* (Michx.) Greene	Fabaceae	*Pinnately*	+	‐
*Eranthis hyemalis* (L.) Salisb.	Ranunculaceae	*Palmately*	‐	‐
*Oxalis debilis* Kunth.	Oxalidaceae	*Palmately*	+	‐
*Petroselinum crispum* (Mill.) Fuss	Apiaceae	*Pinnately*	‐	‐
*Trifolium repens* L.	Fabaceae	*Palmately*	‐	‐
*Ailanthus altissima* (Mill.) Swingle	Simaroubaceae	*Pinnately*	‐	+

## MATERIALS AND METHODS

### Sample collection

Plants were cultivated in the Lilly Greenhouses at Purdue University (West Lafayette, Indiana, USA) (Table 1) in 5 L pots containing commercial potting mix. They were maintained under greenhouse conditions for four to seven months, except *E. hyemalis* and *A. altissima* (Table 1), which were gathered from Horticulture Park on the Purdue University campus in February 2023 and July 2024, respectively. Plants in the greenhouse were watered daily and received weekly applications of liquid fertilizer (Miracle‐Gro, Scotts Company LLC, Marysville, Ohio, USA). Conditions in the glasshouse were maintained under a natural photoperiod and controlled day/night temperatures of 28/22°C, respectively.

### Optical vulnerability curves for rachis and lamina

Vulnerability curves of the rachis and lamina were conducted using the optical vulnerability method and subsequent image analysis that is outlined by Brodribb et al. (2016). Three plants from each herbaceous species were brought to the laboratory, and the roots were carefully washed, or in the case of *A. altissima*, three trees (each were three‐to‐five years old) were cut at the base. The maximum vessel length of *A. altissima* was determined using the air‐injection method (Ewers and Fisher, 1989) from three branches from three individuals and was found to be 132.27 cm ± 12.09 (*n* = 3). Samples of *A. altissima* were immediately recut under water, transported back to the lab, and allowed to rehydrate until leaf water potential was more than –0.5 MPa, measured with a Scholander pressure chamber (PMS International, Albany, Oregon, USA). Plants of herbaceous species were bagged and allowed to rehydrate in a dish of water for at least 2 h until the leaf water potential was more than –0.5 MPa. To assess the vulnerability of the rachis and lamina (and stems in *A. altissima*) simultaneously, leaves and stems were carefully positioned under a microscope (SZM Series: AmScope: Irvine, California, USA) with a digital camera (MU163: AmScope: Irvine, California, USA), so that the lamina and the rachis or petiole (or stem) was in the field of view for all of the images. Once bark had been carefully removed, a glass slide was placed on the stem on which an ultrasound jelly was placed, reducing noise during image analysis. Images of all samples were captured every 5 min. During dehydration of the herbaceous species, neighboring leaves were used to measure water potential using a Scholander pressure chamber every 2 h (Brodribb et al., 2016), starting at hydrated water potentials initially (<–0.5 MPa), and then became increasingly more negative as dehydration progressed. A psychrometer (ICT International: Armidale NSW, Australia) was used to determine water potential during dehydration in *A. altissima* every 10 min. Psychrometers were attached to the main stem, at least 1 m from the cut end, while the sample was still connected to water. Once psychrometer water potential values were greater than –0.5 MPa, samples of *A. altissima* were removed from the water and allowed to dehydrate on the bench. To attach the psychrometer, an area of the branch was carefully scrapped with a razor blade to make the tissue level. The now‐exposed xylem was carefully washed with deionized water, and the area dried with a delicate‐task wipe; the psychrometer was then placed on the exposed xylem and sealed with petroleum jelly.

### Image analysis

Embolism resistance was determined from leaf image stacks, which were then analyzed for embolism events exclusively in the rachis, lamina, or stem. Images were analyzed using ImageJ software following the instructions at GitHub (https://www.opensourceov.org/), and the Open Source Optical Vulnerability (OSOV) toolbox was used to facilitate image analysis (https://github.com/OpenSourceOV/imagej-scripts). Images were imported into the ImageJ software and converted to 8‐bit images. The OSOV toolbox was then used to obtain a stack of differences in pixel threshold across images. We then used the “Analyzed Particle” function to see embolism events in black. To clean stacks more effectively, a modification to the analysis was made during the noise removal portion. The “Filters‐Median” function from the “Process” tab was used to remove any unnecessary noise from the images, accelerating image analysis. Each image stack for the six herbaceous species was processed three times for each replicate, examining embolism events in the whole leaf, then the rachis or petiole and lamina exclusively. A regression between water potential taken from the pressure bomb measurements of the herbaceous species and the time of each image was used to determine the water potential for each image. For *A. altissima* psychrometer, water potentials were fitted against image time; this regression was then used to determine the water potential for each corresponding image. We then obtained the water potential at which 12% (*P*_12_), 50% (*P*_50_), and 88% (*P*_88_) of xylem was embolized in the whole organ, and then each respective tissue (rachis or petiole and lamina) for each replicate to construct means for each species and each corresponding tissue. A mean vulnerability curve for each tissue was then constructed for each species. Each mean vulnerability curve for the corresponding tissue was constructed following Cardoso et al. (2022), in which a mean value was calculated for each 5% increment in embolism formation for each respective tissue in all species measured.

### Statistical analysis

One‐way and two‐way analysis of variences (ANOVAs) were conducted with R version 3.6.1 (R Core Team, 2018) to test for significant differences between the leaflet lamina and the rachis or petiole between the same species (α = 0.05) and between *P*_12_, *P*_50_, and *P*_88_ water potentials. Pairwise comparisons were made with the Tukey Honest Significant Difference test for the difference at *P*_12_, *P*_50,_ and *P*_88_ between the same species (α = 0.05). The “multcompView” function was used to denote letters that indicate significant differences between the leaflet lamina and the rachis or petiole.

## RESULTS

**Figure 1:**
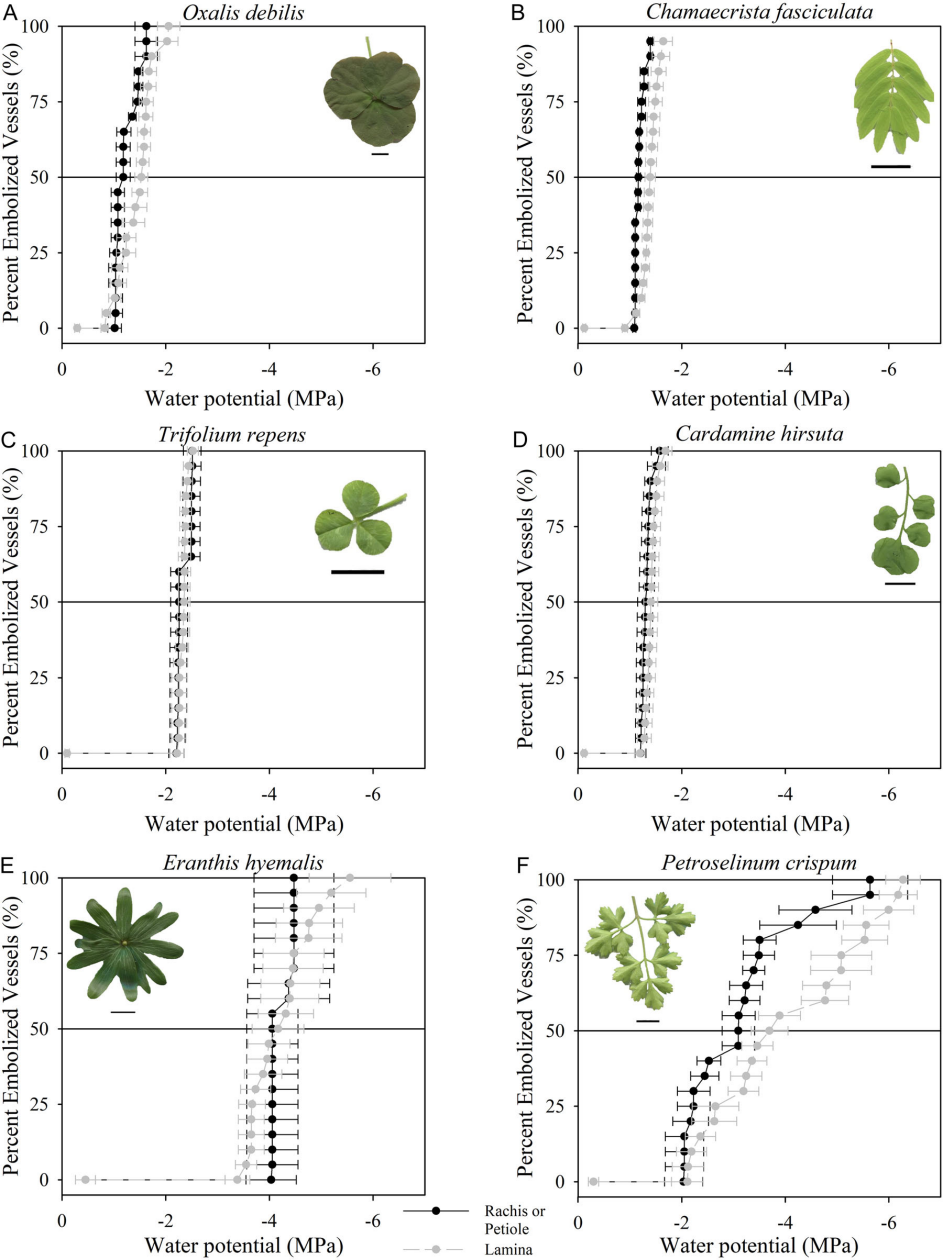
Mean optical vulnerability curves (*n* = 3 biological replicates, ± SE) of the rachis or petiole (black) and the lamina (gray) of six herbaceous species with compound leaves. Representative images of leaves for each species are given, with the scale bar depicting 10 mm. Horizontal lines in all panels denote when 50% of the xylem was embolized. Species with a pulvinus are located at the top of the graph (A and B). Palmately compound species are located on the left side of the figure (A, C, and E), while pinnately compound species are located on the right side of the figure (B, D, and F). The single point in each figure is the mean water potential obtained from the pressure bomb before the sample was removed from the water (*n* = 3, ± SE).

**Figure 2:**
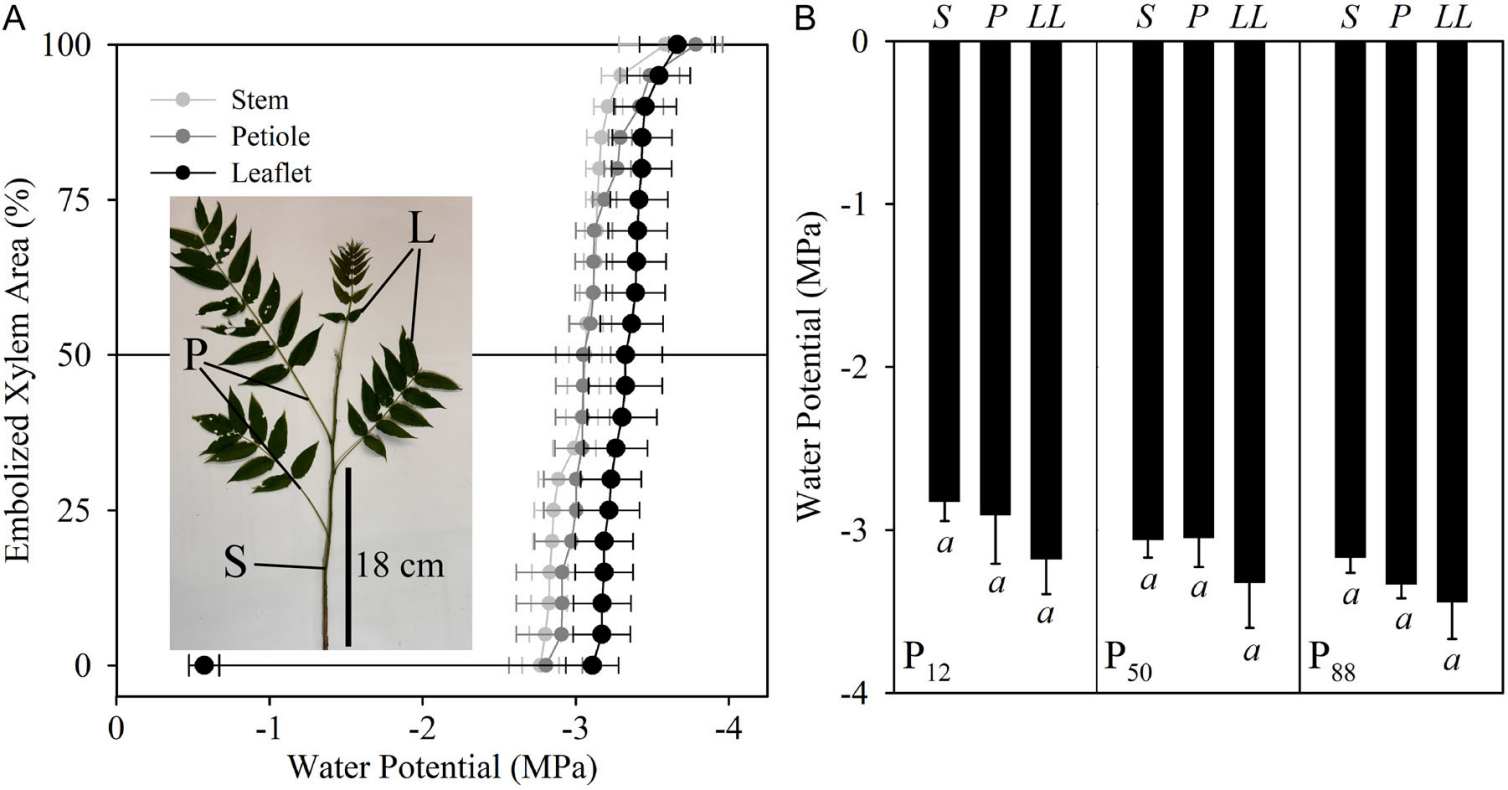
(A) Mean optical vulnerability curves (*n* = 3 biological replicates, ± SE) of the leaflet (black), petiole (dark grey), and stem (light gray) of *Ailanthus altissima*. The solid horizontal line denotes when 50% of the xylem is embolized. (B) Depicts the mean water potential (*n* = 3 ± SE) for the stem (S), petiole (P), and leaflet (LL) when 12% (*P*_12_), 50% (*P*_50_), and 88% (*P*_88_) of the xylem is embolized, respectively. Significant differences in *P*_12_, *P*_50_, and *P*_88_ between tissues are denoted with lowercase italicized letters. The single point in each figure is the mean water potential obtained from the pressure bomb before the sample was removed from the water (*n* = 3, ± SE).

**Figure 3:**
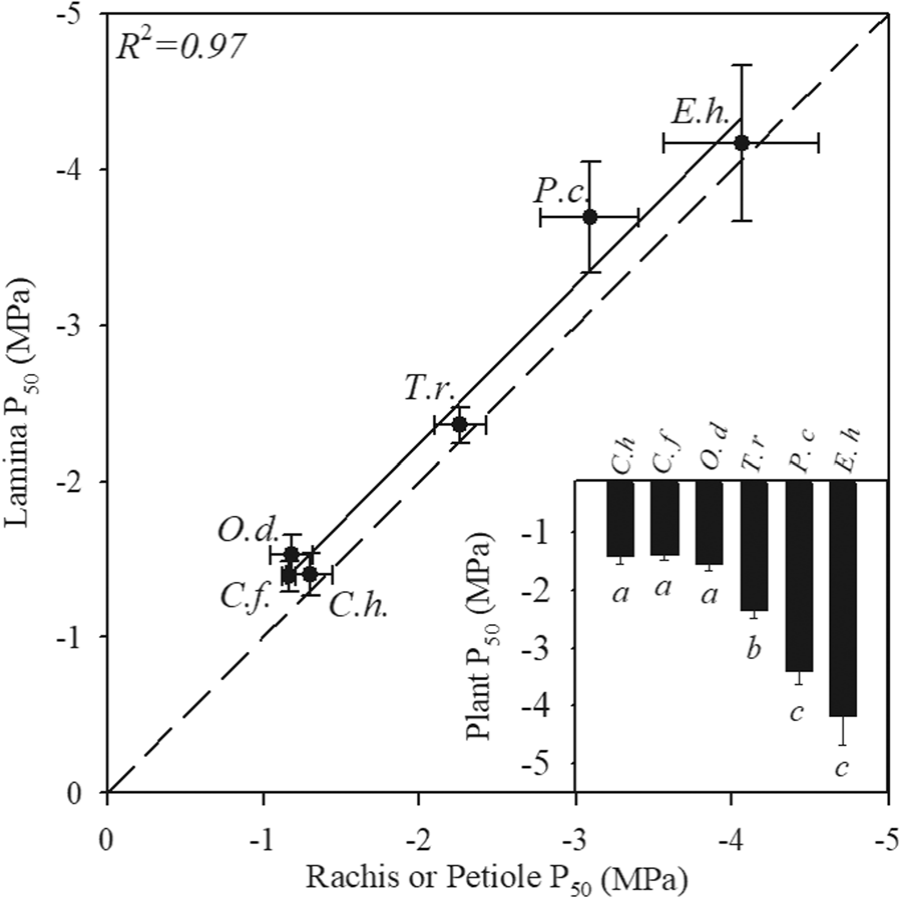
The relationship between the water potential at which 50% of the xylem was embolized (*P*_50_) in the lamina and the rachis or petiole across six herbaceous species with compound leaves (*n* = 3, ± SE). The black dashed line represents a 1:1 relationship. The solid black line represents a significant linear regression. The figure inserted in the lower right‐hand corner illustrates the mean whole leaf *P*_50_ across the six herbaceous species with compound leaves (*n* = 3, ± SE). Significant differences in mean whole leaf *P*_50_ are denoted with italicized letters above each corresponding error bar. Abbreviations are given for each species *E.h*. is *Eranthis hyemalis*, *P.c*. is *Petroselinum crispum*, *T.r*. is *Trifolium repens*, *O.d*. is *Oxalis debilis*, *C.h*. is *Cardamine hirsuta*, and is *C.f. Chamaecrista fasciculata*.

**Figure 4:**
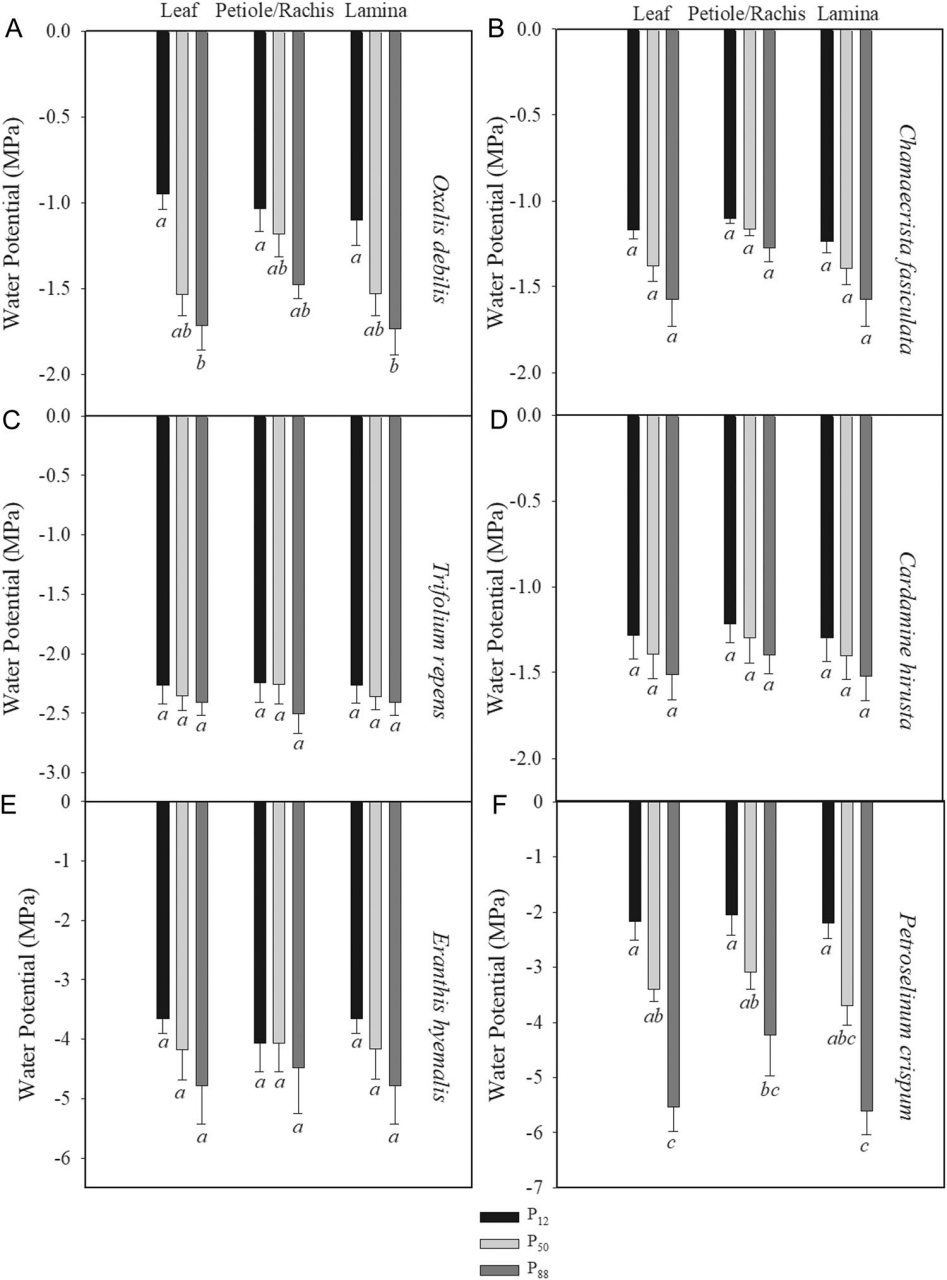
Mean water potential at which 12% (*P*_12_, black), 50% (*P*_50_, light gray), and 88% (*P*_88_, dark gray) of the xylem was embolized for the whole leaf, petiole or rachis, and the leaf lamina of all six herbaceous species measured. Significant differences are denoted in lowercase italicized letters below each standard error bar.

We found no evidence of hydraulic segmentation across the compound leaves of the six herbaceous angiosperm species (Figure 1), or the compound leaves of the deciduous tree *A. altissima* (Figure 2). The average difference between the *P*_50_ of the lamina and corresponding rachis or petiole across all herbaceous species was a nonsignificant 0.27 ± 0.12 MPa (*n* = 18, ± se, t.test = 0.45). The average difference between the *P*_50_ of the leaf lamina and the corresponding petiole of *A. altissima* was 0.17 ± 0.48 MPa and was not significantly different across replicates (t.test = 0.40). A significant relationship was found between the *P*_50_ of the rachis or petiole and the corresponding lamina in all of the herbaceous species examined (*R* ^2^ = 0.97) (Figure 3). Furthermore, we found no significant difference at each *P*_12_, *P*_50,_ and *P*_88_ value of embolism formation across every region of the leaf in any of the herbaceous species (Figure 4, ANOVA) or the deciduous tree *A. altissima* (Figure 2, ANOVA).

The most embolism‐resistant species measured was *E. hyemalis*, with a mean whole leaf *P*_50_ of –4.18 ± 0.51 (*n* = 3, ± se) (Figure 3). The most vulnerable species measured was *O. debilis*, with a mean whole leaf *P*_50_ of –0.95 ± 0.09 (*n* = 3, ± 3). The two herbaceous species with the most embolism resistant xylem (*E. hyemalis* and *P. crispum*) had the greatest variation in embolism resistance between replicates, but this did not result in a significant difference between the embolism resistance of the petiole or rachis and corresponding lamina within individual leaves (Figure 4). There was no difference in mean embolism resistance between palmately and pinnately compound‐leaved herbaceous angiosperm species (*t* = 0.46, df = 2, *P* = 0.34).

**Figure 5:**
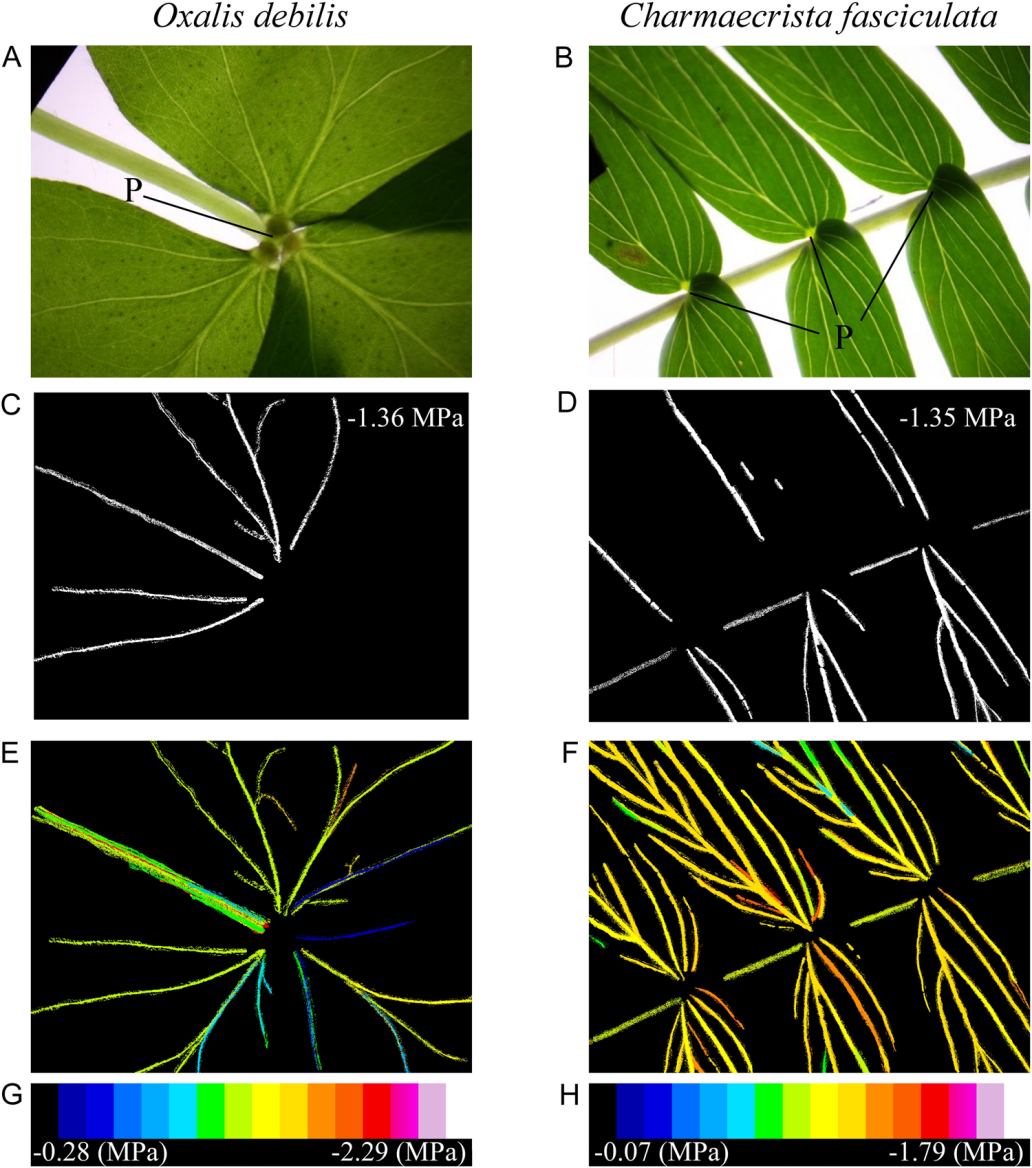
Representative images of an *Oxalis debilis* (A) and *Chamaecrista fasciculata* (B), with a black arrow pointing to the pulvini in each respective image. Images of a leaf in which a single embolism event that propagated through the pulvini is shown in color (C and D) with the corresponding water potential at which the embolism events occurred. All embolism events across the leaf were color‐coded by the water potential at which they were observed (E and F). For each species, a color scale (G and H) depicting the water potential with each embolism event that occurred is shown below the corresponding image.

Our results suggest that in two herbaceous angiosperm species, a pulvinus in the leaves does not act as a barrier to embolism spread from the rachis or petiole to the lamina. During image analysis, we observed that single embolism events were able to readily propagate across the pulvinus in the leaves of both *O. debilis* and *C. fasciculata*, spanning both the rachis or petiole and veins of the corresponding leaflet lamina (Figure 5). In both of these species, we observed the first embolism events occurring in the rachis or petiole of the compound leaves and the final embolism events occurring in the minor veins of the leaflets (Figure 5). In both species, embolism events were capable of spreading into multiple leaflets (Figure 5). We did not notice any difference in the timing of embolism formation between individual leaflets across the compound leaves of any species assessed. In the deciduous tree *A. atltissima*, we found that the presence of an abscission zone at the base of the leaflet did not result in within‐leaf hydraulic segmentation (Figure 2). Using the air injection method (Ewers and Fisher, 1989), we could find no evidence of vessels traversing the stem into the petiole, or from petiole into the leaf lamina in *A. altissima*, *O. debilis*, and *C. fasciculata*.

## DISCUSSION

### Hydraulic segmentation between leaflet lamina and the rachis or petiole is not common

We found that hydraulic segmentation across compound leaves (Song et al., 2022) is not universal in angiosperms, and may indeed be rare (Figures 2, 3). We did not observe differences in the timing of embolism formation across the leaves of six herbaceous angiosperms or one deciduous tree measured here. The lack of hydraulic segmentation across the seven diverse angiosperm species suggests that the observations of variation in embolism resistance across the leaves of *Fraxinus mandshurica* and *Juglans mandshurica* (Song et al., 2022) are not generalizable for all angiosperms. It seems likely that nonhydraulic factors, at least not related to the spreading of embolism across the leaf, have played an equally, if not more important role in the evolution of compound leaves in angiosperms (Nicotra et al., 2011).

In herbaceous plants, hydraulic segmentation between organs (stems and leaves) is observed once secondary xylem develops in the stem (Pereira et al., 2018; Dória et al., 2018, 2019; Haverroth et al., 2024). Given that the vascular tissue in leaves is composed of primary xylem (Růžička et al., 2015), differences in xylem anatomy are unlikely to drive variation in embolism resistance across tissues. We do not yet know the mechanism that causes hydraulic segmentation to occur in the compound leaves of some species but not others. Explanations for hydraulic segmentation across compound leaves include differences in xylem anatomy contributing to variation in vessel diameter, vessel connectivity, pit membrane area, and vessel tapering or widening, which have been suggested to play substantial roles in driving differences in embolism resistance within and between species (Scholz et al., 2013; Guan et al., 2021; Kaack et al., 2021; Mrad et al., 2021; Isasa et al., 2023). Similar to previous studies (Scoffoni and Jansen, 2016; Hochberg et al., 2019; Brodribb et al., 2021), we observed that the xylem of the midrib of leaflets and the petiole or rachis was the first to experience embolism in the angiosperm species, while minor veins typically embolized last.

### Embolism can readily spread through a pulvinus

Our observations indicate that nonrandom vessel endings at junction points, like abscission zones or pulvini, which have been attributed to causing hydraulic segmentation between leaves and stems (Klepsch et al., 2018; Guan et al., 2021), do not consistently inhibit embolism spread within compound leaves. We did not find variation in embolism resistance between the leaflet lamina and the rachis in the pinnately compound leaves of *A. altissima*, a deciduous tree that has abscission zones at the base of each leaflet, like the leaves of *Fraxinus* and *Juglans* (Song et al., 2022). We were also interested in testing whether the pulvinus, a joint‐like structure at the base of leaflets that facilitates nyctinasty (Nakata and Takahara, 2022), could prevent embolism spread. The results from *O. debilis* and *C. fasciculata* suggest that the pulvinus in the leaves of these species does not act as a barrier to embolism spread from the rachis or petiole to the lamina (Figure 3). We also found that air could not be injected through the xylem across nodes from the stem into the petiole, or from the petiole into the leaflet of *O. debilis*, *C. fasciculata*, and *A. altissima* suggesting that vessels did not traverse the pulvinus or the abscission zone of these species.

### Consequences for drought tolerance strategies in vegetative tissue

In compound‐leaved deciduous tree species, Song et al. (2022) hypothesized that considerable hydraulic segmentation across the leaf provides an additional hydraulic fuse during drought that could protect stems from embolism formation in distal leaflets, with leaflets experiencing embolism first, followed by rachis, then the stem. This adaptation not only protects stems from embolism spreading from leaves during drought but also greatly reduces leaf area, and thus transpiration, during drought. The shedding of leaflets during a drought is commonly observed across compound‐leaved trees (Brodribb and Holbrook, 2003; Pineda‐García et al., 2013; Liu et al., 2015; Wolfe et al., 2016; Rehschuh et al., 2020), and also in some herbaceous species like those in the genus *Chamaecrista* (Luisa Martínez and Moreno‐Casasola, 1998), and species in the genus *Ailanthus* (Schall and Davis, 2009). The absence of hydraulic segmentation across the compound leaves of angiosperm species observed here suggests that embolism is unlikely to be the primary mechanism driving leaflet shedding in these species. Rather, the abscission of leaflets is most likely actively driven by endogenous metabolic signals and independent of embolism formation in the xylem. Our results support the prevailing view that in herbaceous species in which secondary growth has not yet developed, there is limited variation in embolism resistance across the plant (Skelton et al., 2017). It seems that not all compound‐leaved angiosperm species invest in more vulnerable leaflets and embolism‐resistant stems as a drought‐tolerance strategy.

### Variation in leaf embolism resistance across herbaceous species

Considerable variation in mean leaf embolism resistance was found across the seven angiosperm species examined (Figures 1, 2). The two most embolism‐resistant herbaceous species, *E. hyemalis* and *P. crispum*, displayed the greatest variation in embolism resistance between replicate leaves. Cardoso et al. (2020) found that as mean leaf embolism resistance increases across species, so too does the variation in embolism resistance between leaves. Our data suggest that embolism formation in some species can occur over a wide range of water potentials and may not be entirely pressure‐driven (Guan et al., 2021). Evidence for this comes from the significant differences in P_88_ between the petiole or rachis and the leaflet lamina in these two species, which could be due to variation in the speed of embolism propagation once high levels of embolism are sustained in the xylem. *Eranthis hyemalis* commonly grows through extensive winter frost events, having leaves that can tolerate at least –11°C (Lundquist and Pellett, 1976). Given our results of considerable leaf embolism resistance in this species, it is tempting to hypothesize a potential link between freezing tolerance and leaf embolism resistance in geophytic herbaceous species; an observation that has been made before in trees (Wang et al., 2022; Yin et al., 2022; Hartill et al., 2023; Volaire et al., 2023).

The variation we observed in embolism resistance across the six herbaceous species examined in this study is considerable and mirrors previous work that indicates that while many herbaceous species have highly vulnerable xylem, some species have relatively embolism‐resistant xylem (Lens et al., 2016). Many families that are dominated by herbaceous species, including Oxalidaceae, Apiaceae, and Brassicaceae, are underrepresented in global analyses of embolism resistance (Venturas et al., 2017; Dória et al., 2019; Lens et al., 2022). The optical vulnerability method provides one of the few means to assess leaf embolism resistance across herbaceous species. Our finding that there is no variation in embolism resistance between leaflet lamina and petioles in compound‐leaved herbaceous species suggests that future studies could validly observe either of these tissues and generate functionally informative leaf vulnerability curves.

## CONCLUSIONS

In this study, we sought to determine if variation in embolism resistance between leaflets and the petiole or rachis of compound leaved angiosperm species was a rule and whether a pulvinus or abscission zone is capable of inhibiting embolism spread from rachis or petiole into the lamina. We found that pulvini and abscission zones do not stop embolism from spreading across a compound leaf (Figure 5) and that there was no significant difference in embolism resistance across the parts of compound leaves of any of the herbaceous species (Figure 4) or the deciduous tree examined (Figure 2). We did observe substantial variation in embolism resistance among the six herbaceous species sampled (Figure 3), suggesting that further work is needed to examine the anatomical and ecological drivers of variation in embolism resistance across herbaceous species.

## AUTHOR CONTRIBUTIONS

Both authors designed the study; I.R. collected and analyzed vulnerability curves and wrote the manuscript with help from S.M.

## Data Availability

The data associated with the findings in this study are available at Dryad (https://datadryad.org/stash/dataset/doi:10.5061/dryad.8pk0p2nxc).
